# Supplementary material for: Deep Chemical and Physico-Chemical Characterization of Antifungal Industrial Chitosans—Biocontrol Applications
Source: Molecules. 2023 Jan 18;28(3):966. doi: 10.3390/molecules28030966 (PMC9919833; doi:10.3390/molecules28030966)
Supplement: Supplementary file 1 [file molecules-28-00966-s001.zip › molecules-2094814-supplementary.pdf]

Supplementary information

Deep chemical and physico-chemical characterization of antifungal industrial chitosans - Biocontrol applications

Gaël Huet, Yunhui Wang, Christian Gardrat, Daphnée Brulé, Amélie Vax, Cédric Le Coz, Frédérique Pichavant, Silvère Bonnet, Benoit Poinssot and Véronique Coma *

*Corresponding author

*Correspondence: veronique.coma@u-bordeaux.fr (V.C.)

Table of contents

^{1}H NMR : 2

SEC MALS 12

XPS 13

TGA 16

Electrospray 21

MALDI-TOF 22

Pyrogram 23

Bioactivity 25

^{1}H NMR :

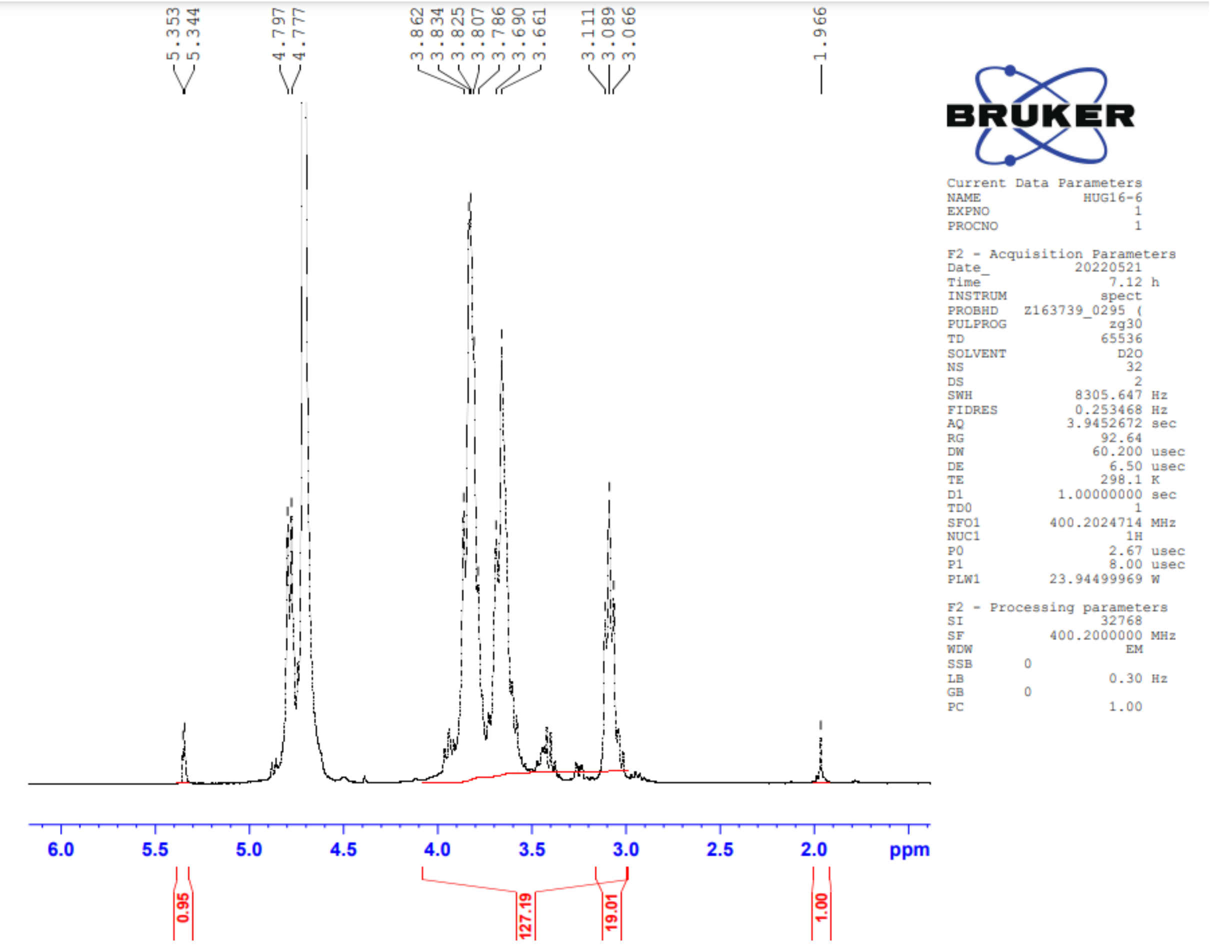

Figure S1 : Liquid-state ^{1}H NMR spectrum of CHI-1 : DA = 98%.

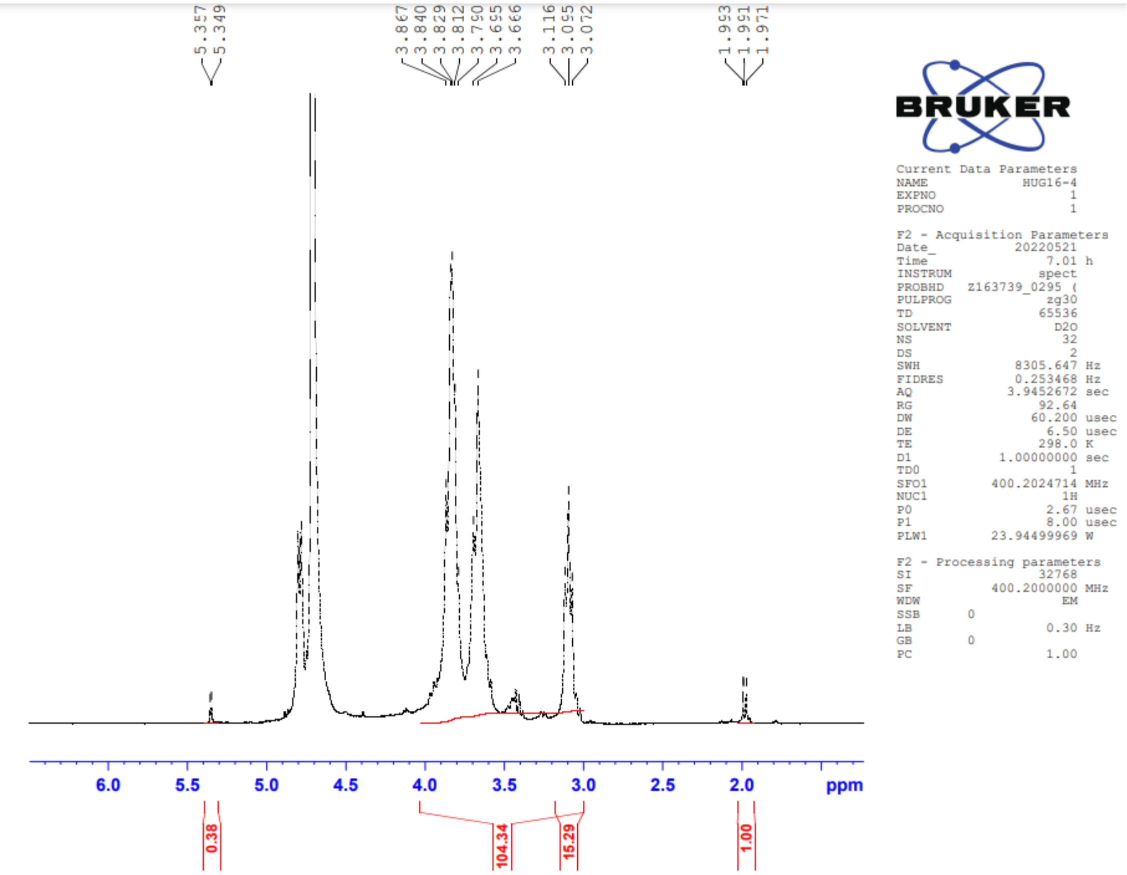

Figure S2 : Liquid-state ^{1}H NMR spectrum of CHI-2 : DA = 98%.

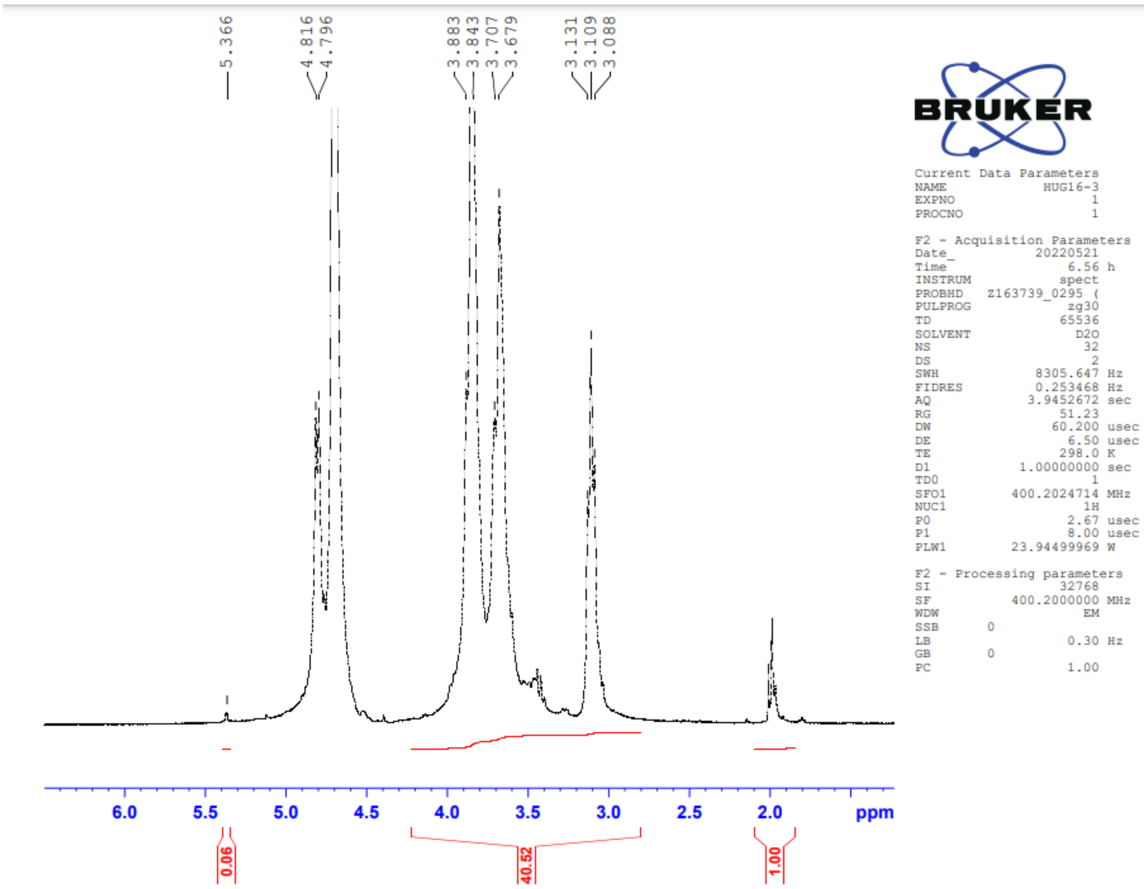

Figure S3 : Liquid-state ^{1}H NMR spectrum of CHI-3 : DA = 95%.

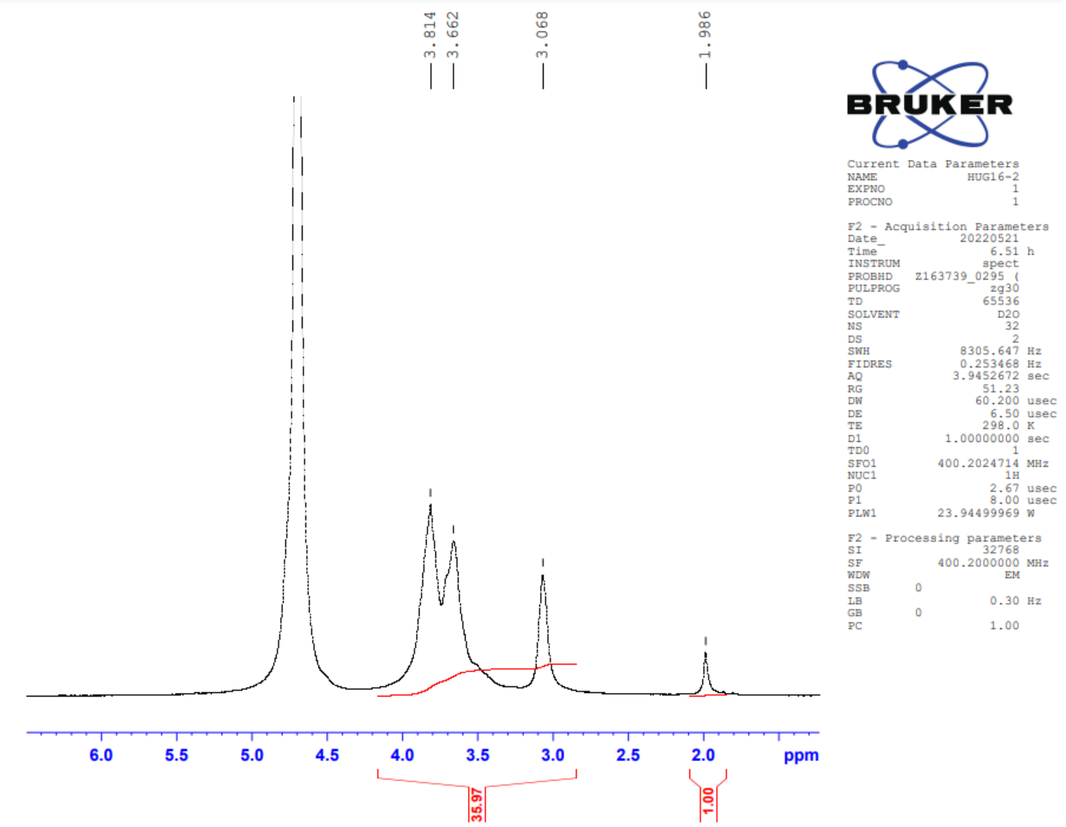

Figure S4 : Liquid-state ^{1}H NMR spectrum of CHI-4 : DA = 94%.

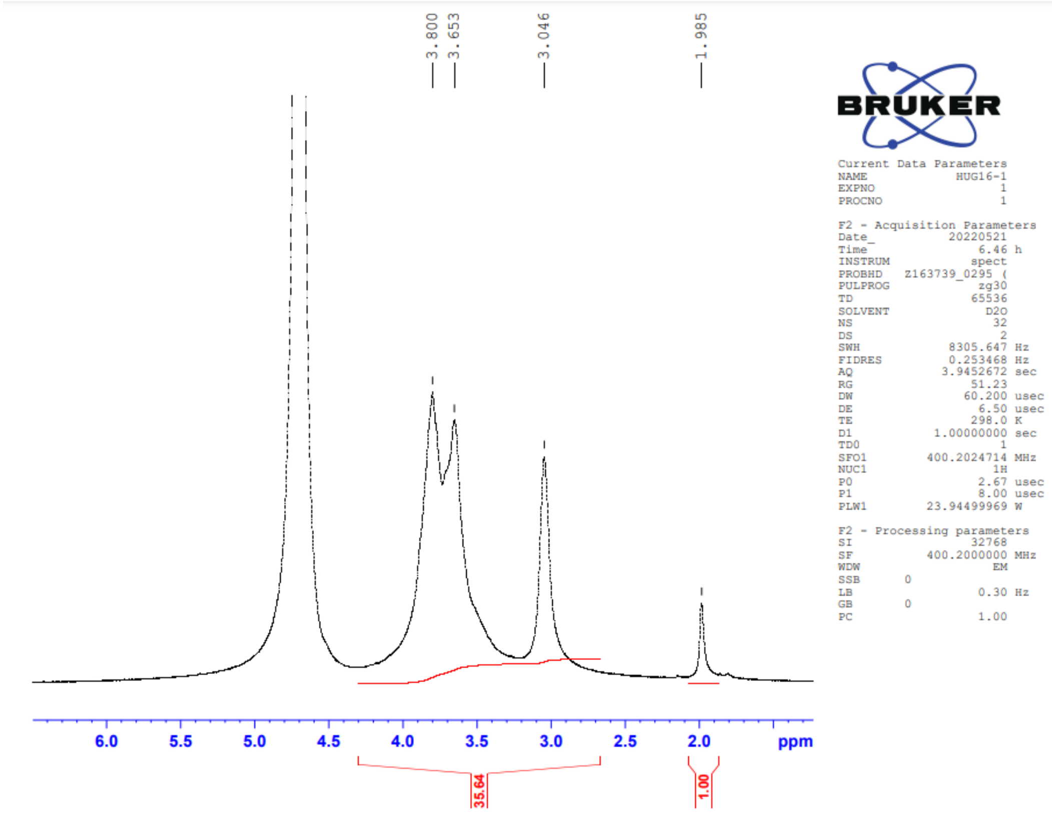

Figure S5 : Liquid-state ^{1}H NMR Spectrum of CHI-5 : DA = 93%.

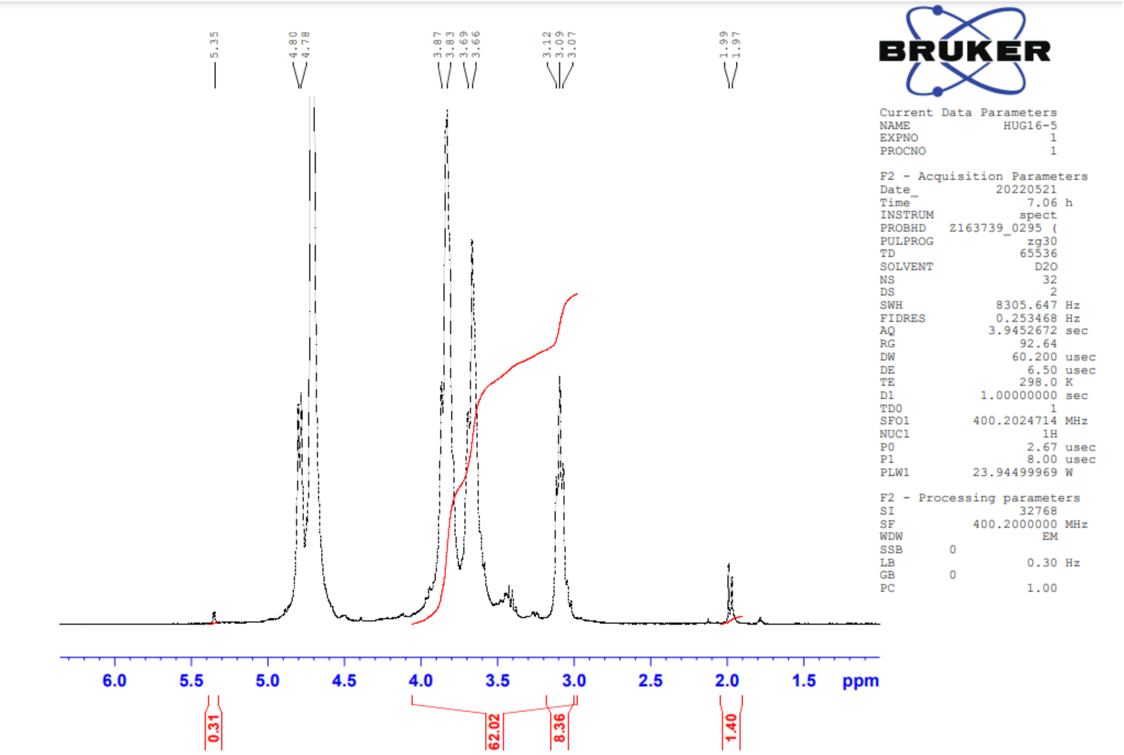

Figure S6 : Liquid-state ^{1}H NMR Spectrum of a DP50 chitosan at Room temperature and solubilized overnight : DP = 54

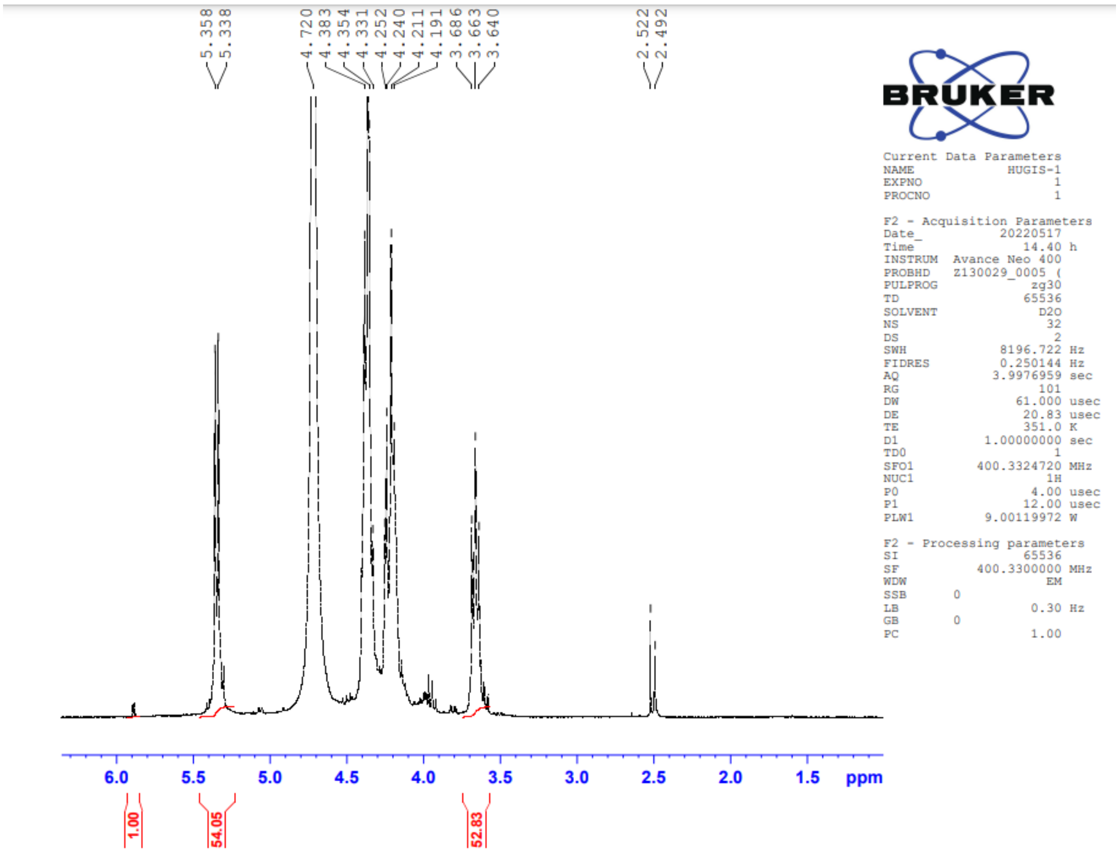

Figure S7 : Liquid-state ^{1}H NMR Spectrum of a DP50 chitosan at 80°C and solubilized for 6h and stabilized for 20 minutes : DP = 54

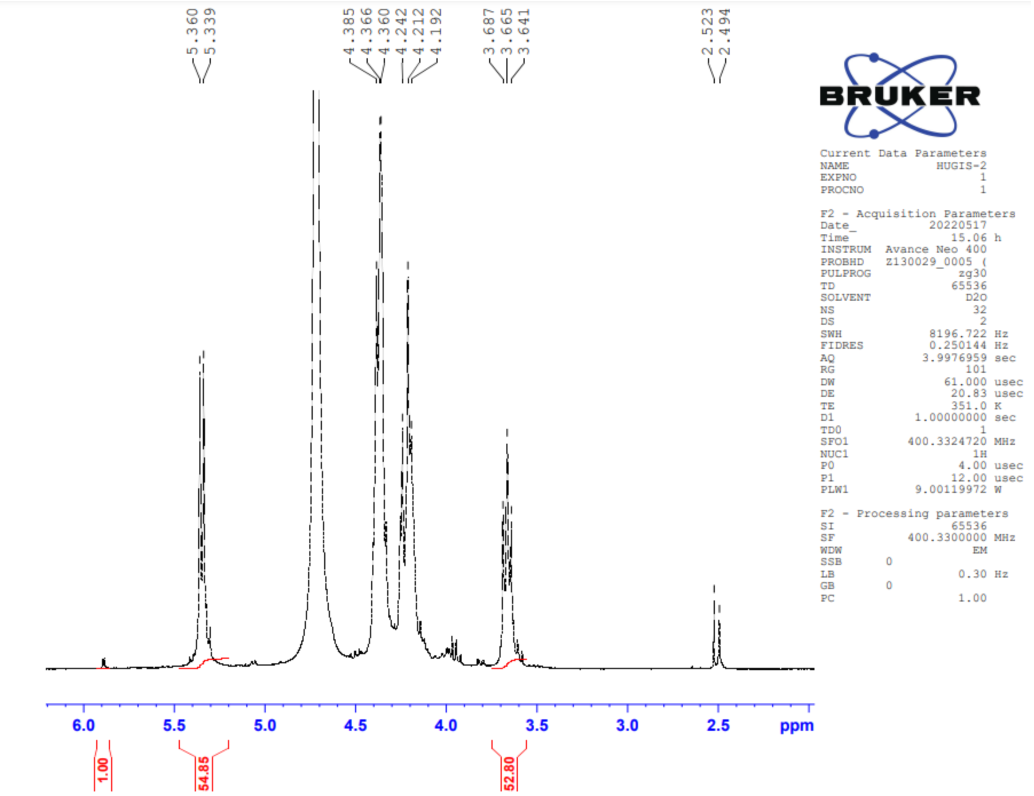

Figure S8 : Liquid-state ^{1}H NMR Spectrum of a DP50 chitosan at 80°C and solubilized overnight and stabilized for 20 minutes : DP = 54

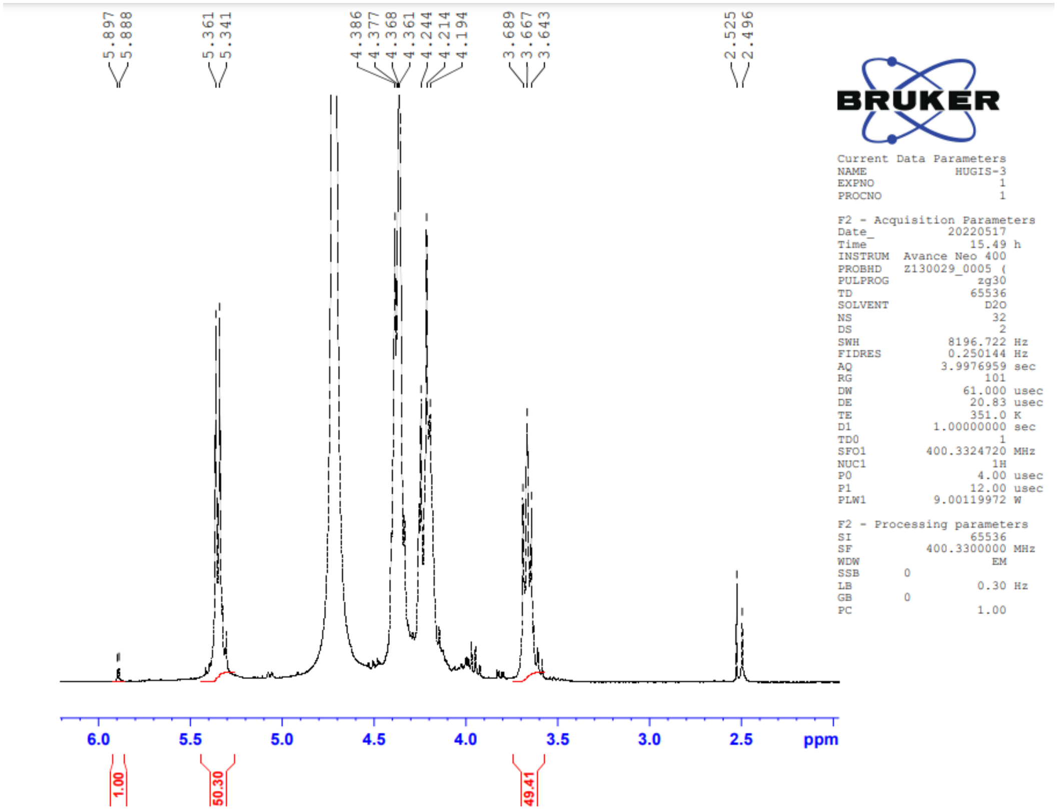

Figure S9 : Liquid-state ^{1}H NMR Spectrum of a DP50 chitosan at 80°C and solubilized overnight and stabilized for 40 minutes : DP = 50

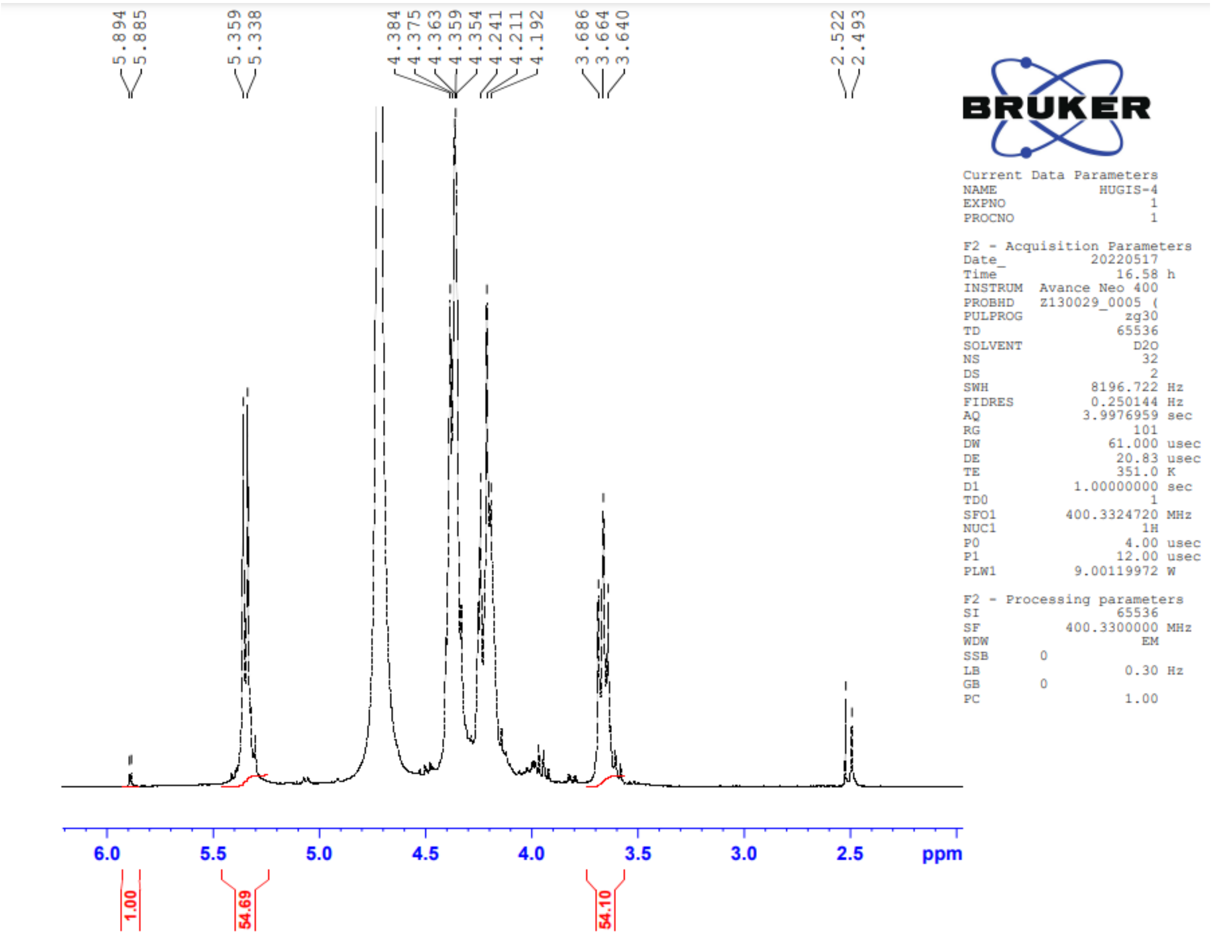

Figure S10 : Liquid-state ^{1}H NMR Spectrum of a DP50 chitosan at 80°C and solubilized overnight and stabilized for 60 minutes : DP = 54

SEC MALS

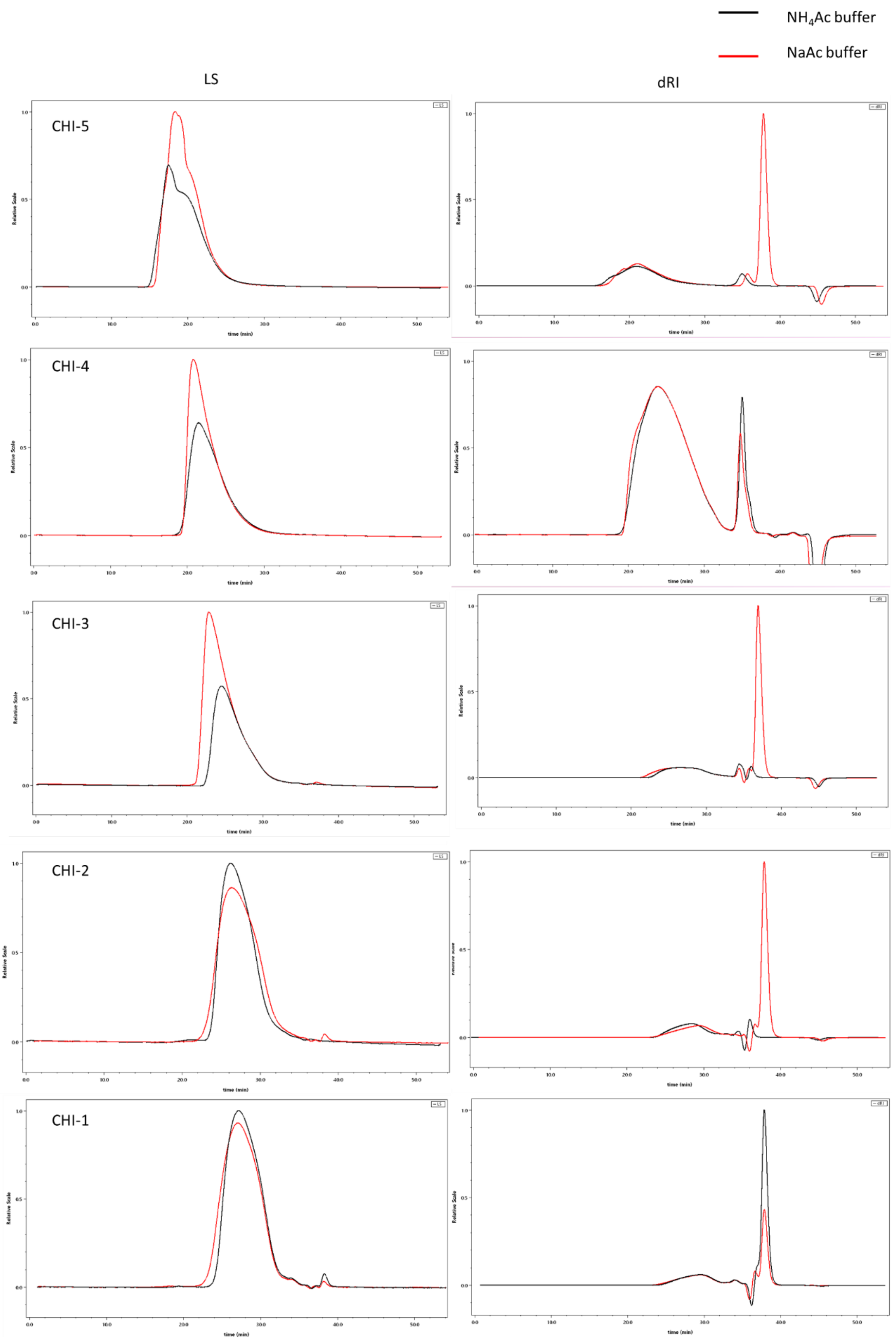

Figure S11 : SEC MALS chromatograms for CHI-1, CHI-2, CHI-3 CHI-4 and CHI-5 on two G4000PWXL and G3000PWXL gel columns in NH_4Ac buffer (black) and NaAc buffer (red). The black and red curves are LS (90° angle) and dRI responses, respectively.

Echantillon	dn/dc	
	Sodium acetate	Ammonium acetate
CHI-5	0.1742	0.1808
CHI-4	0.1727	0.1844
CHI-3	0.1609	0.1711
CHI-2	NA	0.1763
CHI-1	0.1677	0.1663

Table S1 : dn/dc determined for the different chiitosans in sodium acetate buffer and ammonium acetate buffer.

XPS

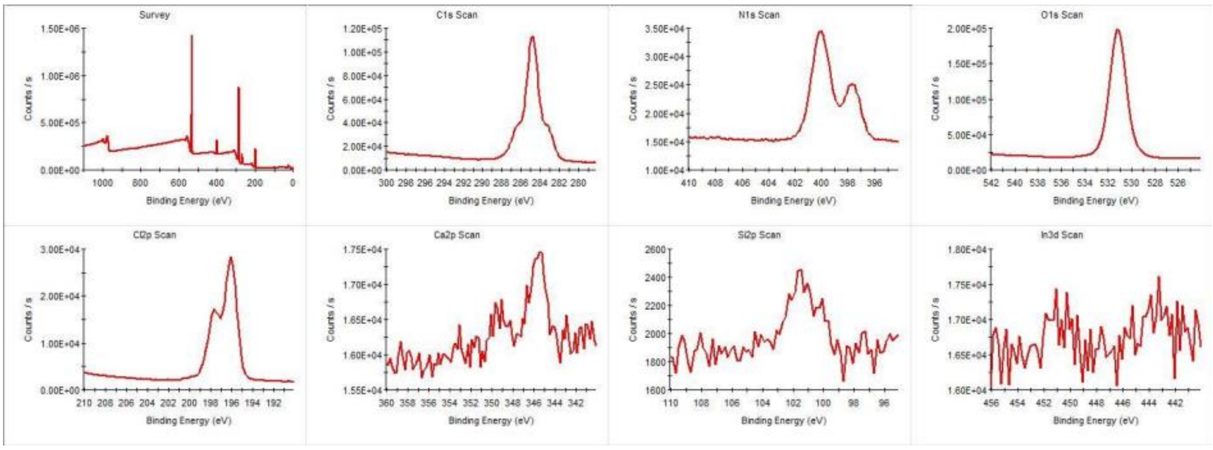

Figure S12 : XPS results from CHI-1.

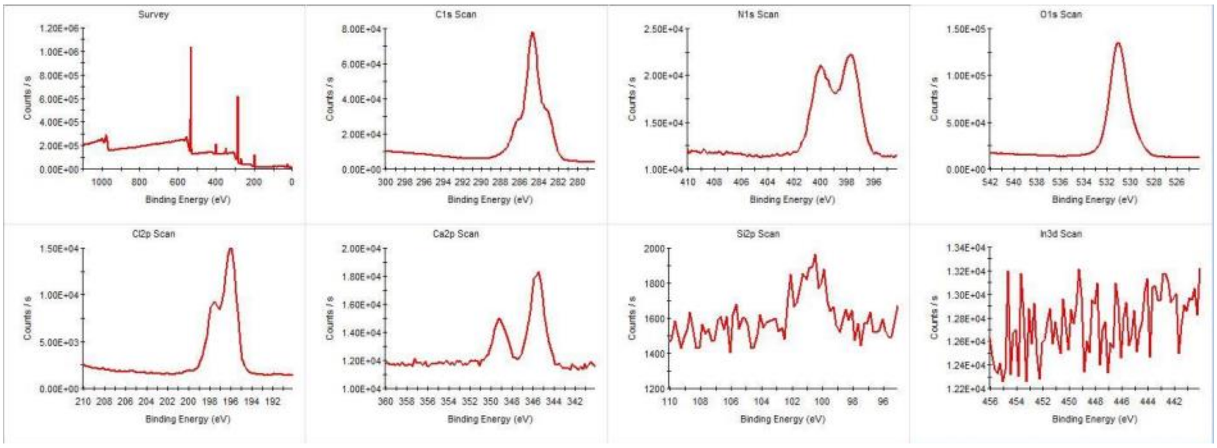

Figure S13 : XPS results from CHI-2.

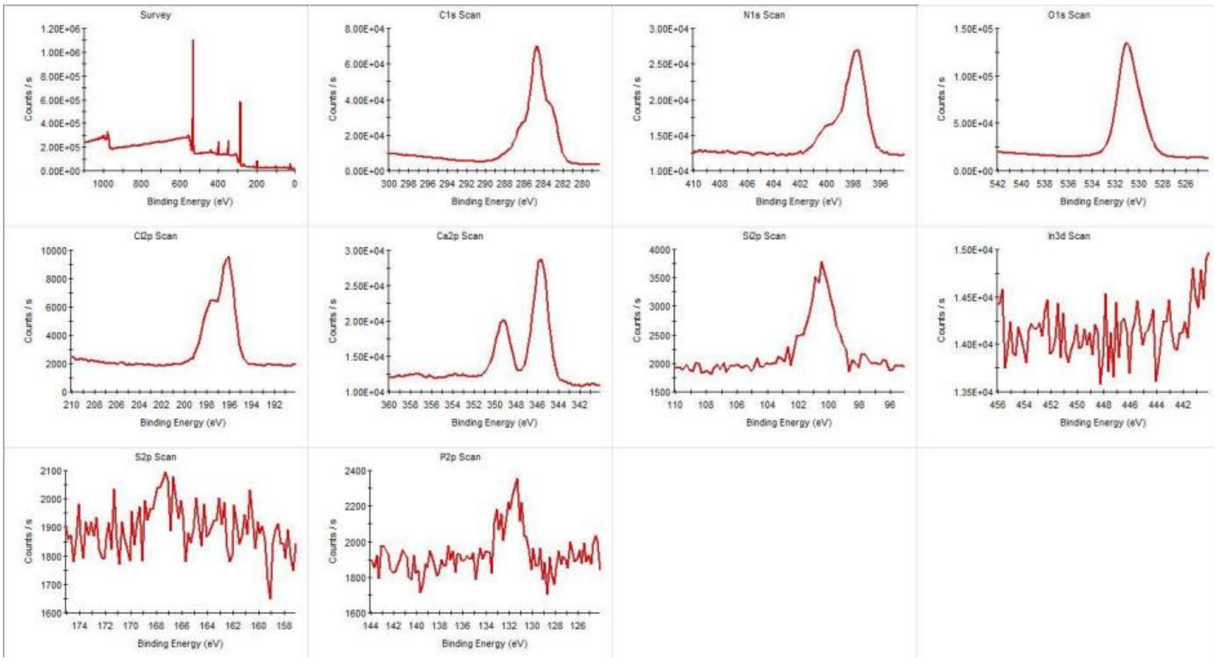

Figure S14: XPS results from CHI-3.

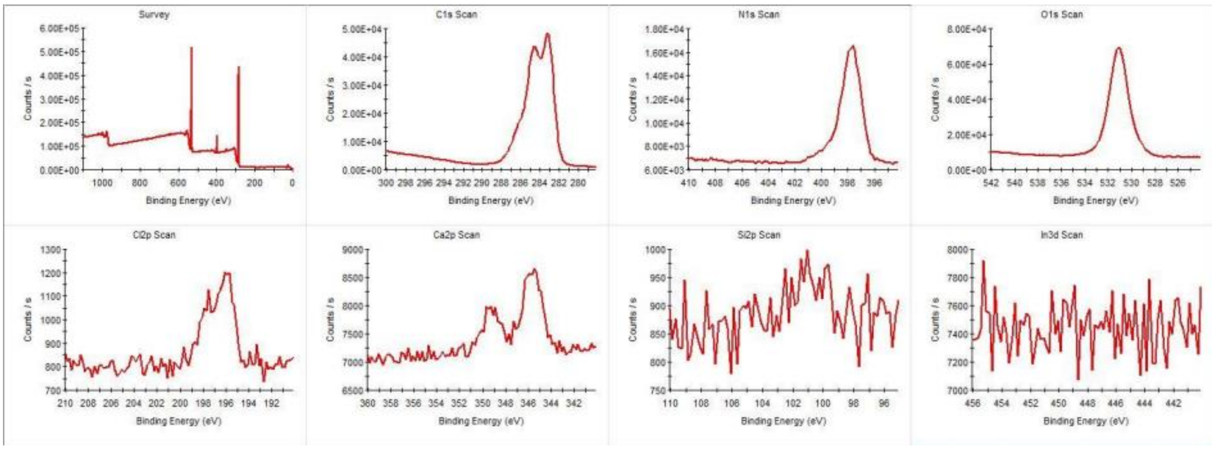

Figure S15 : XPS results from CHI-4.

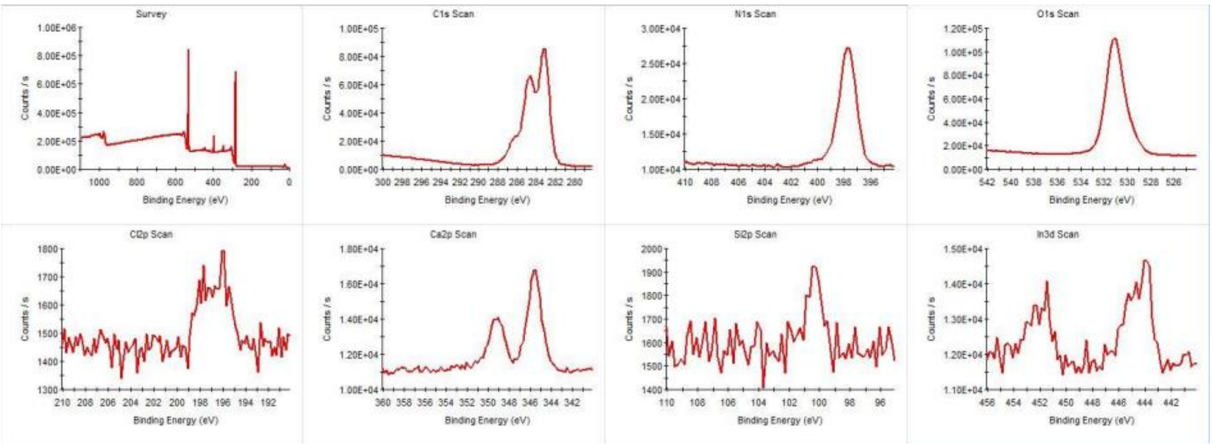

Figure S16 : XPS results from CHI-5.

TGA

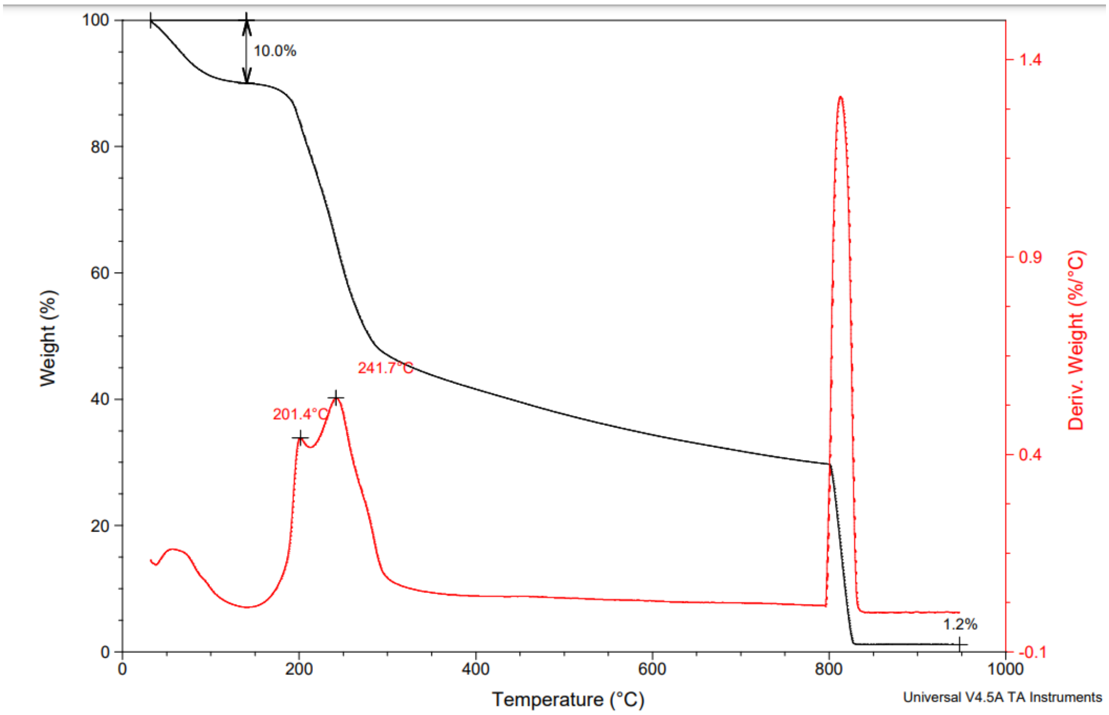

Figure S17 : Thermogravimetric and differential thermal analysis of under nitrogen (till 800°C) then air (800-950°C) of CHI-1.

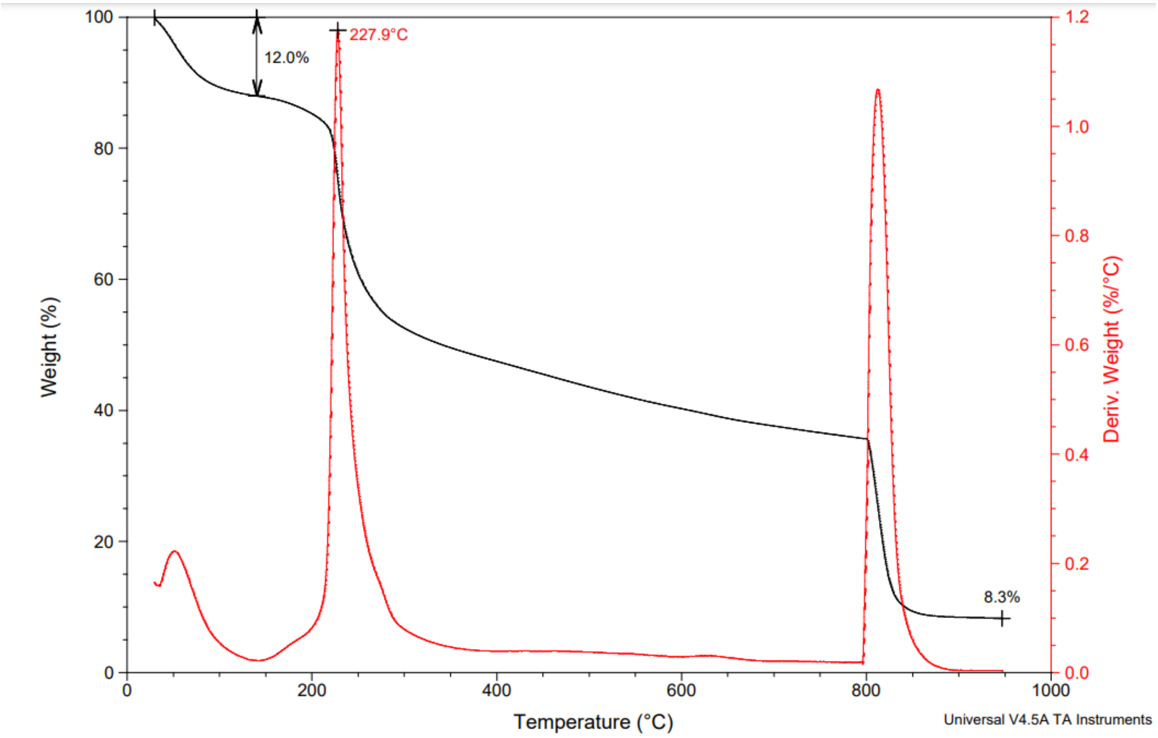

Figure S18 : Thermogravimetric and differential thermal analysis of under nitrogen (till 800°C) then air (800-950°C) of CHI-2.

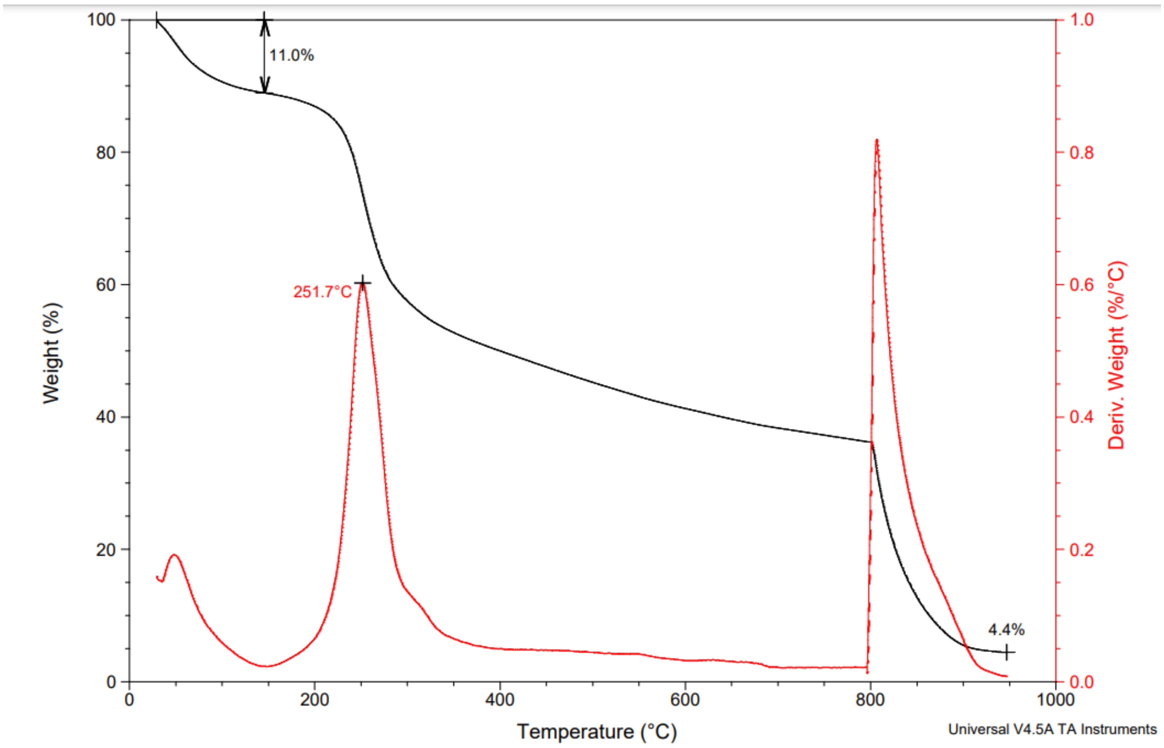

Figure S19 : Thermogravimetric and differential thermal analysis of under nitrogen (till 800°C) then air (800-950°C) of CHI-3.

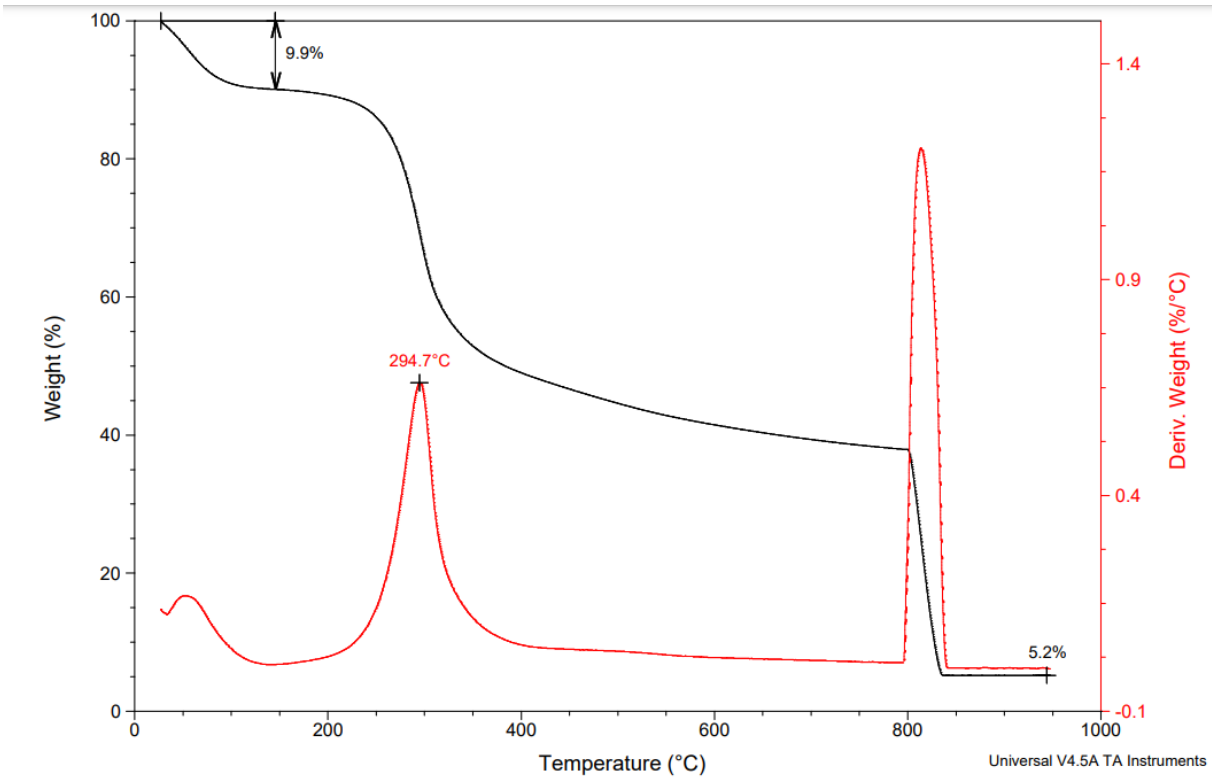

Figure S20 : Thermogravimetric and differential thermal analysis of under nitrogen (till 800°C) then air (800-950°C) of CHI-4.

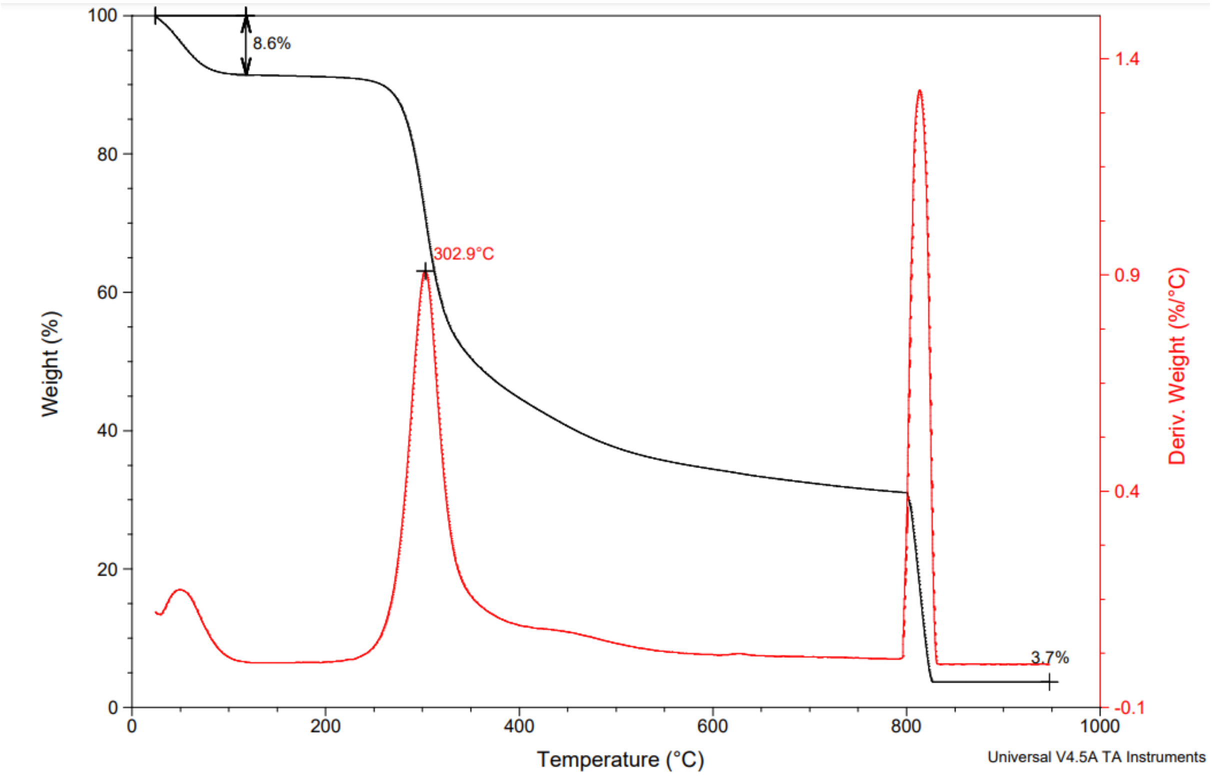

Figure S21 : Thermogravimetric and differential thermal analysis of under nitrogen (till 800°C) then air (800-950°C) of CHI-5.

Electrospray

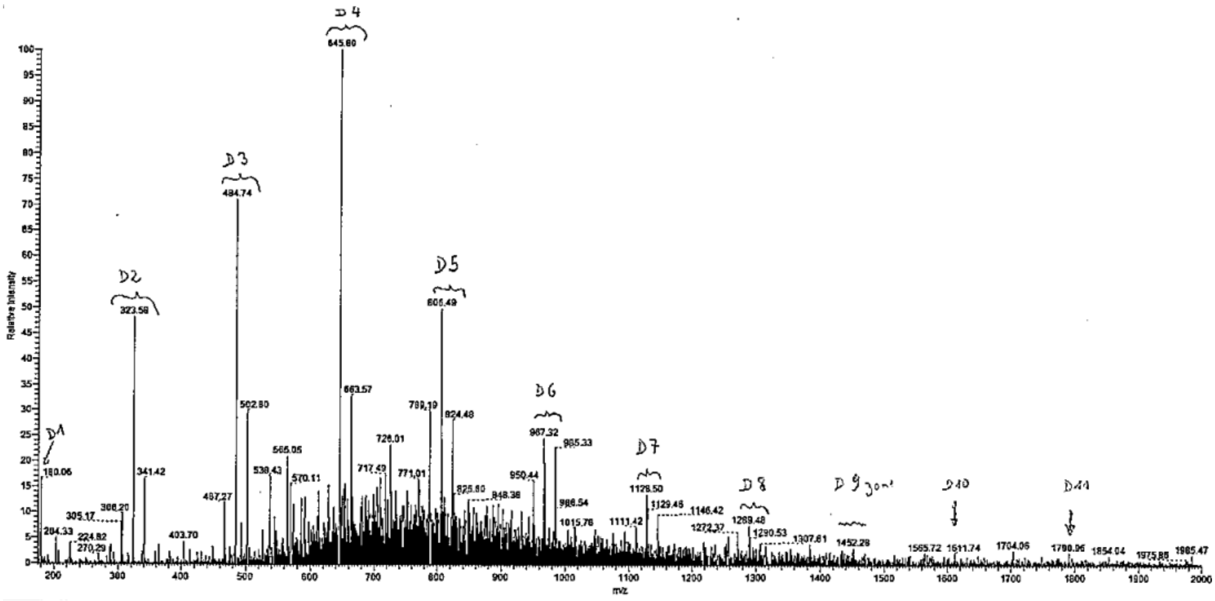

Figure S22 : Electrospray (+) mass spectrum of CHI-2

MALDI-TOF

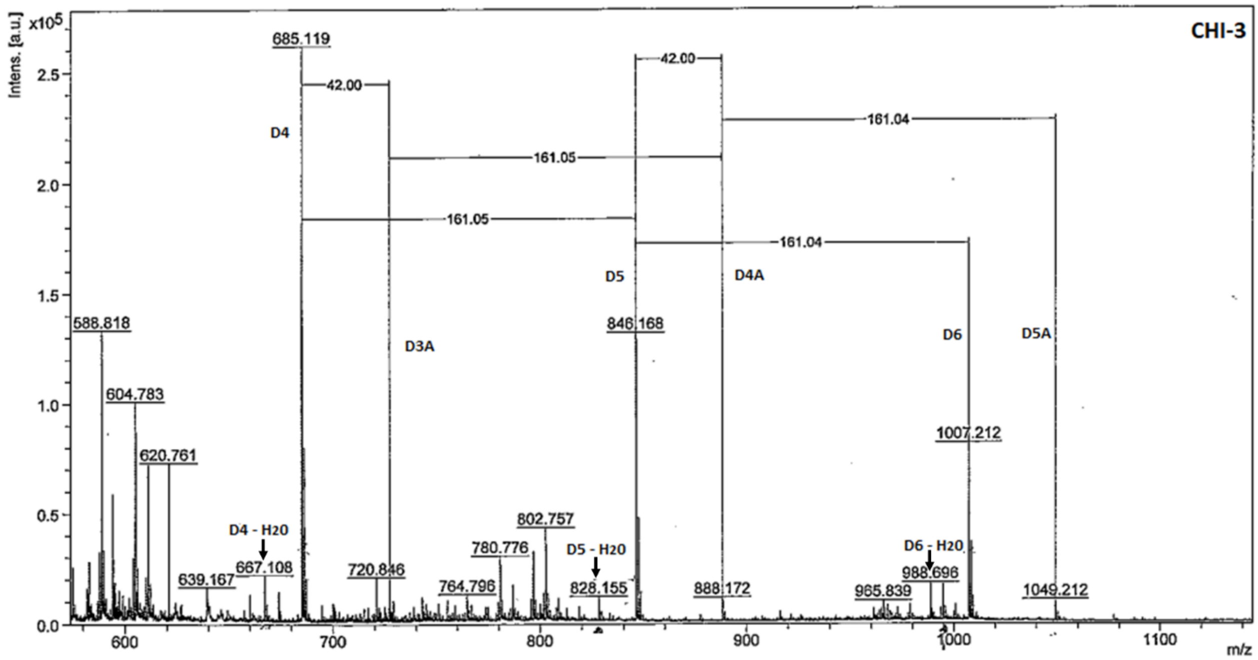

Figure S23 : MALDI-TOF (+) mass spectrum of CHI-3 (reflectron mode)

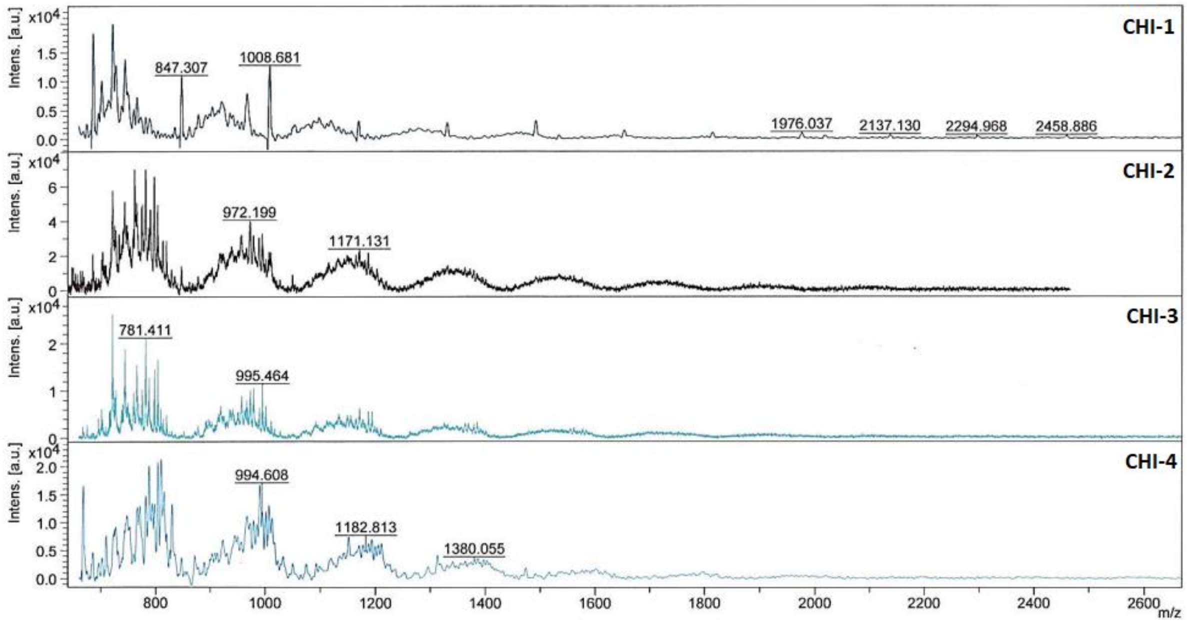

Figure S24 : MALDI-TOF (+) mass spectra of CHI-1 to CHI-4 (linear mode)

Pyrograms

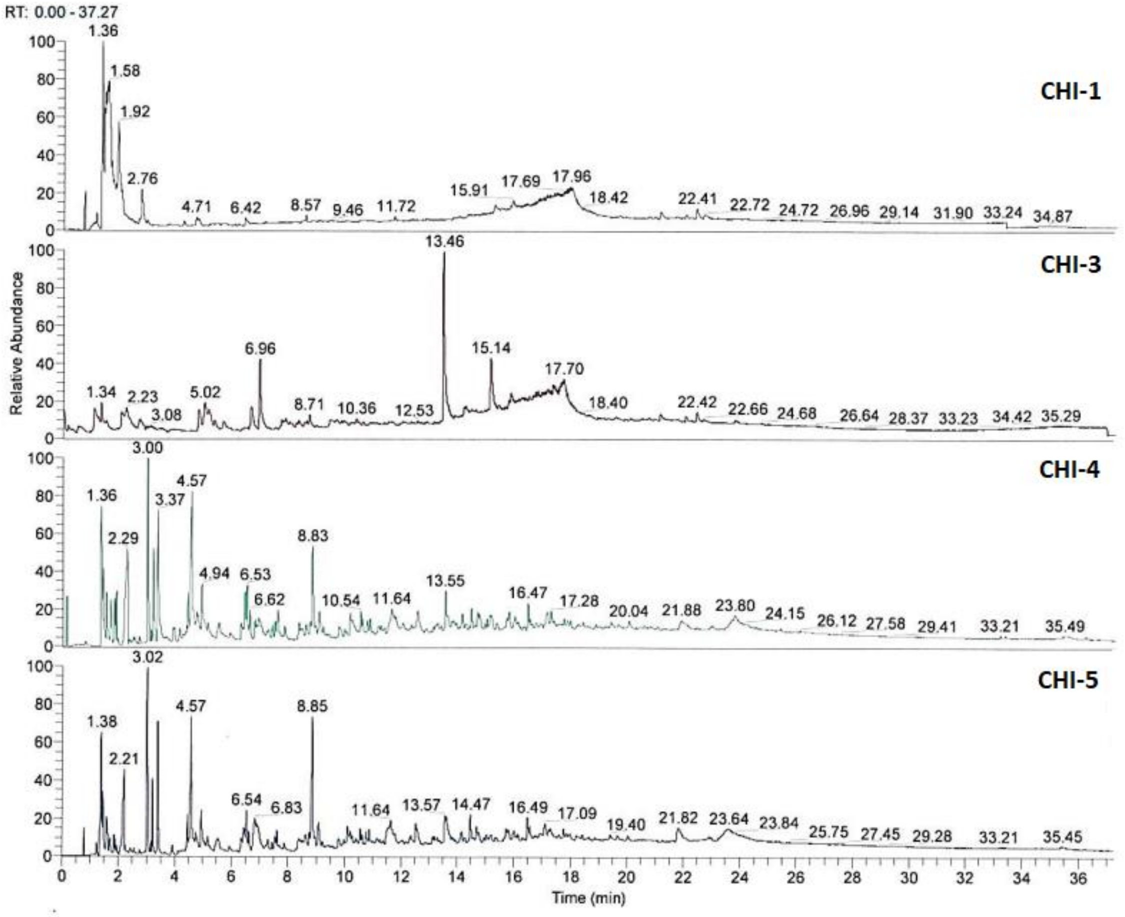

Figure S25 : Py-GC/MS of CHI-1, CHI-3, CHI-4 and CHI-5.

NIST proposal	Retention time t_R (min)					References
	CHI1	CHI-2	CHI-3	CHI-4	CHI-5	
Carbone dioxide<br>Hydrogen chloride<br>2-Methylfurane<br>Acetic acid	1.36<br>1.58<br>1.92<br>-	1.21<br>1.23<br>1.78	1.34<br>-<br>-	1.36<br>-<br>1.91<br>2.29	1.38<br>-<br>-<br>2.21	g,h<br><br>j<br>b,h,i,j,l
2,5-Dimethylfurane	2.76	2.62	-	2.73	2.74	h
Pyrazine	-		-	3.00	3.02	b,c,d,f,h
Pyridine	-	-	-	3.19	3.21	c,d,f,h,j,l
Pyrrole	-	-	-	3.37	3.39	f,h,j,l
2- or 3-Methylpyridine	-	-	-	4.46	4.46	c,l
Methylpyrazine	-	-	-	4.57	4.57	a,b,c,d,e,f,h
2-cyclopentene-1-one	-	-	-	4.78	4.75	h,l
2-Methyl-(1H)-pyrrole	-	-	-	4.94	4.95	h
3-Methyl-(1H)-pyrrole	-	-	-	5.16	5.17	f,h,j,l
2-Methyl-2-cyclopentene-1-one	6.42	6.25	-	6.33	6.34	h,j
2-(5H)-Furanone	-	-	-	6.46	6.44	h
Ethyl pyrazine	-	-	-	6.53	6.54	b,c,d,e,f,h
2,3-Dimethylpyrazine	-	-	-	6.62	6.62	a,b,c,d
2,4-Dimethyl-(1H)-pyrrole	-	-	-	6.80	-	h
6-Methyl-4-(1H)-pyrimidinone	-	7.74	-	7.86	7.87	l
2-Ethyl-5-methyl-pyrazine	-	-	-	8.36	8.38	c
Acetylpyrazine	-	-	-	8.83	8.85	b,d,f,h
2,3-Dimethylcyclopentene-1-one	-	-	-	9.21	9.20	l
Acetylpyrrole	-	-	-	9.78	9.80	c,d,e
Indol-5-ol	-	-	-	10.54	10.56	
Dimethyl-2-pyrrole carbonitrile	-	-	-	10.60	10.62	
1-(5-Methylpyrazinyl)-1-ethanone	-	-	-	10.79	10.81	
Acetyl-6-methylpyrazine	-	-	-	10.87	10.89	
Pyridine-3-ol	-	-	-	11.64	11.66	k
1H-Pyrrolo[2,3-b]-pyridine	-	-	-	12.57	12.59	
4-Hydroxy-(1,7/1,8) -naphthyridine<br>RN: 54569-29-8<br>RN: 54920-82-0	-	13.52	13.46	13.57	13.59	
Indole	-	-	-	14.15	14.17	j
2-Acetamino-5-methyl-imidazole	-	-	-	14.48	14.50	h,l
5-Methylbenzimidazole	-	-	-	14.70	14.72	
4-Aminobenzyl cyanide	-	-	-	14.76	14.78	
4-Methyl-naphthyridine	-	-	-	14.95	14.97	
3-Acetoxy-2(1H)-pyridone	-	-	-	15.03	15.03	
Amino-4-hydroxyquinoline	-	-	-	15.12	15.14	
2-Hydroxy-7-methyl-1,8-naphthyridine	-	15.17	15.14	15.18	15.20	
2,4,6-Trimethyl-benzonitrile	-	-	-	15.72	15.74	
7-Methyl-1H-indole	-		15.86	15.81	15.83	
3-(1H-Pyrazol-4-yl)-pyridine	-	-	-	16.47	-	

Table S2: Identified compounds generated by the pyrolysis of chitosans CHI-1 to CHI-5

(a) D. Knorr, T.P. Wampler & R.A. Teutonico (1985). Formation of pyrazines by chitin pyrolysis. Journal of Food Science, 50, 1762-1763. Doi: 10.1111/j.1365-2621.tb10589.x
(b) C.-K. Shu (1998). Degradation products formed from glucosamine in water. Journal of Agricultural and Food Chemistry, 46, 1129-1131. Doi: 10.1021/jf970812n
(c) J. Chen & C.-T. Ho (1998). Volatile compounds formed from thermal degradation of glucosamine in a dry system. Journal of Agricultural and Food Chemistry, 46, 1971-1974. Doi: 10.1021/jf971021o
(d) J. Chen, M. Wang & C.-T. Ho (1998). Volatile compounds generated from thermal degradation of *N*-acetylglucosamine. Journal of Agricultural and Food Chemistry, 46, 3207-3209. Doi: 10.1021/jf980129g
(e) L. Zeng, C. Qin, L. Wang & W. Li (2011). Volatile compounds formed by pyrolysis of chitosan. Carbohydrate Polymers, 83, 1553-1557. Doi: 10.1016/j.carbpol.2010.10.007
(f) I. Corazzari, R.Nistico, F. Turci, M.G. Faga, F. Fransozo, S. Tabasso, G. Magnacca (2015). Advanced physico-chemical characterization of chitosan with FTIR and GCMS: Thermal degradation and water adsorption capacity. Polymer Degradation and Stability, 112, 1-9. Doi: 10.1016/j.polymdegradstab.2014.12.006.
(g) Y. Qiao, S. Chen, Y. Liu, H. Sun, S. Jia, J. Shi, C.M. Pedersen, Y. Wang, & X. Hou (2015). Pyrolysis of chitin biomass: TG-MS analysis and solid char residue characterization. Carbohydrate Polymers, 133, 163-170. Doi: 10.1016/j.carbpol.2015.07.005.
(h) C. Liu, H. Zhang, R. Xiao & S. Wu (2017). Value-added organonitrogen chemicals evolution from the pyrolysis of chitin and chitosan. Carbohydrate Polymers, 156, 118-124. Doi: 10.1016/j.carbpol.2016.09.024.
(i) H.F.G. Barbosa, D.S. Francisco, A.P.G. Ferreira & E.T.G. Cavalheiro (2019). A new look towards the thermal decomposition of chitins and chitosans with different degrees of deacetylation by coupled TG-FTIR. Carbohydrate Polymers, 225, 115232. Doi: 10.1016/j.carbpol.2019.115232.
(j) P. Zhang, H. Hu, H. Tang, Y. Yang, H. Liu, Q. Lu, X. Li, N. Worasuwannarak & H. Yao (2019). In-depth experimental study of pyrolysis characteristics of raw and cooking treated shrimp shell samples. Renewable Energy, 139, 730-738. Doi: 10.1016/j.renene.2019.02.119.
(k) M. Nikahd, J. Mikusek, L.-J. Yu, M.L. Coote, M.G. Banwell, C. Ma & M.G. Gardiner (2020). Exploiting chitin as a source of biologically fixed nitrogen: Formation and full characterization of small-molecule hetero- and carbocyclic pyrolysis products. The Journal of Organic Chemistry, 85, 4583-4593. Doi: 10.1021/acs.joc.9b03438.
(l) P. Wang & Y. Shen (2022). Catalytic pyrolysis of cellulose and chitin with calcinated dolomite – Pyrolysis kinetics and products analysis. Fuel, 312, 122875. Doi: 10.1016/j.fuel.2021.122875.

Bioactivity

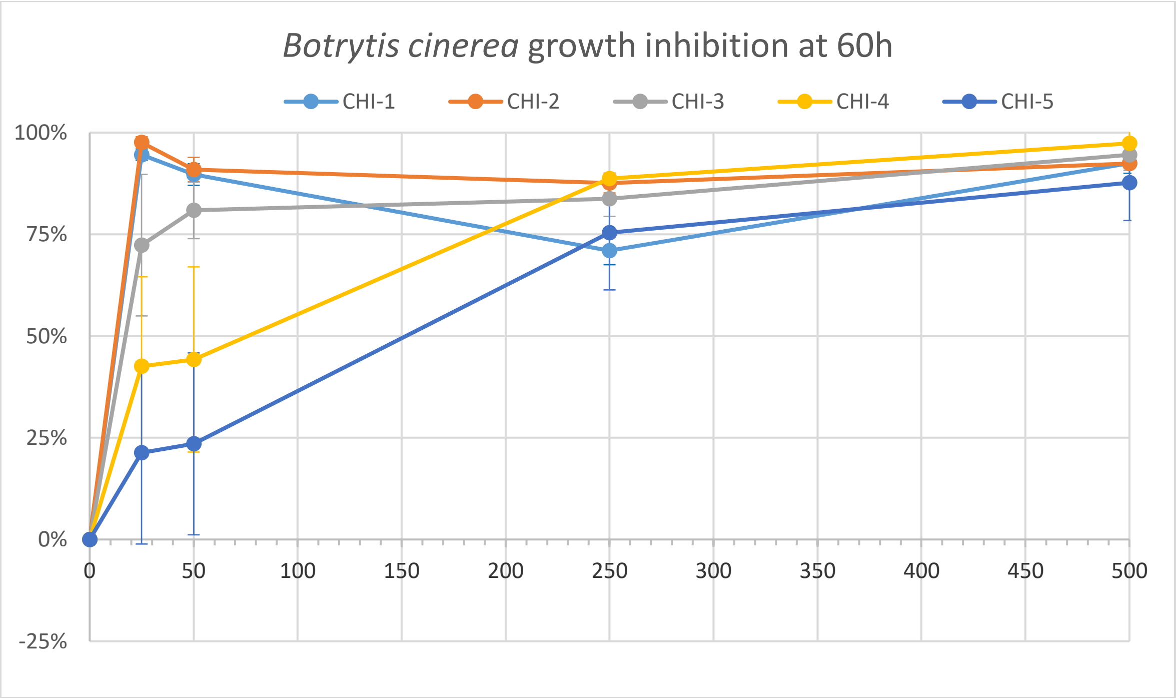

Figure S26 : Growth inhibition of *Botrytis cinerea in vitro* after CHI-1 to -5 treatment. *B. cinerea* conidia (2.10^5/mL) were treated with various concentrations of CHI-1 to CHI-5 (25, 50, 250 and 500 mg/L) and mycelial growth was followed by optical density at 492 nm measured by the microplate reader Bioscreener up to 60 hpt. Values represent the mean of three independent experiments ± SE (n=9).
